# Untargeted Metabolomic Profiling Identifies Breed-Specific Metabolomic Signatures in the Longissimus Dorsi Meat of Beichuan White Goats and Tianfu Goats

**DOI:** 10.3390/metabo16080537

**Published:** 2026-07-30

**Authors:** Kun Du, Kaisen Zhao, Yisong Wang, Ting He, Jinchan Luo, Xiaofeng Liu, Fanglong Li, Jian Yang, Ming Fu, Daihua Wang

**Affiliations:** 1 Animal Disease Prevention and Control and Healthy Breeding Engineering Technology Research Centre, Mianyang Normal University, Mianyang 621000, China; dukun1672@163.com; 2 Bureau of Agriculture and Rural, Tongjiang County, Bazhong 636700, China; zhaofl0303@163.com (K.Z.); wyswonder@163.com (Y.W.); 19198960069@163.com (T.H.); luojinchan98@163.com (J.L.); liuxiaofeng0619@163.com (X.L.); 13648186420@163.com (F.L.); 15280966856@163.com (J.Y.); fm13881666428@126.com (M.F.)

**Keywords:** Beichuan White goat, Tianfu goat, longissimus dorsi meat, metabolomics, difference

## Abstract

Background: Local goat resources have unique meat quality and phenotypic advantages over cultivated breeds. Clarifying breed-specific metabolic differences in meat helps understand meat quality formation and resource utilization. Methods: the study used ultra-high performance liquid chromatography coupled with tandem mass spectrometry (UHPLC-MS/MS) technology to perform untargeted metabolomic analysis on the longissimus dorsi meat of Beichuan White goats (BCWG) and Tianfu goats (TMG). Results: A total of 452 differential metabolites were identified between the two breeds, including 100 lipids and lipid-like molecules, 74 organic acids and their derivatives, 12 nucleosides, nucleotides, and analogues, 204 organic compounds, and 62 unclassified metabolites. Among them, 132 metabolites exhibited higher abundance in Beichuan White goats and 320 metabolites showed significantly higher abundance in Tianfu goats. Metabolomic profiling revealed that differential metabolites of lipids and amino acids (e.g., valine and glutamate) might be correlated with interbreed disparities in meat sensory quality and flavor, as indicated by metabolomic profiling. BCWG displayed relatively higher abundances of nucleoside and nucleotide metabolites, which might hint at a possible link to variations in disease-resistance capacity. Among the 12 nucleoside and nucleotide differential metabolites, UDP-N-acetylglucosamine, 1-methyllinosine, 1-methylguanosine, 3-methylcytidine, and N-(2-Furanylmethyl) adenosine could serve as candidate molecular indicators for studying immune-related traits in goats. Conclusions: The study clarifies the breed-specific metabolic characteristics of the longissimus dorsi meat in BCWG and TMG at the metabolite level, provides novel molecular markers for chevon quality evaluation and immune trait selection, and lays a theoretical foundation for the rational development and utilization of local goat resources and improvement of cultivated meat goat breeds.

## 1. Introduction

China is a major producer of chevon, with its production accounting for a quarter of the world’s total production [1]. Chevon plays an important role in the human diet because of its high protein, low fat content, and low cholesterol [2]. Many research studies have showed that the skeletal muscle weight of sheep is close to 40% of its body weight [3,4]. With improvements in living standards, consumer demand for high-quality chevon has continuously increased, and chevon, thanks to its favorable meat quality, desirable flavor, and rich nutritional value, has gradually become a popular choice among consumers [5]. Beichuan White goat is a local goat breed mainly used for meat production. It is native to Beichuan County, Sichuan Province, and is also distributed across adjacent counties (districts) such as Maowen, Pingwu, and Songpan. It has the advantages of good production performance, a high reproductive rate, and strong adaptability, etc. As the first synthetic meat goat breed in China, the Tianfu goat was bred by crossing native Sichuan goats with outstanding foreign goat varieties (e.g., Boer and Nubian goats). This provincially certified cultivated breed features strong adaptability, high roughage resistance, and fast growth, and has therefore been extensively farmed in Sichuan, Henan, Jiangsu, and other regions of China [6]. It has no direct origin relationship with the Beichuan White goat.

Metabolites represent the terminal downstream products of cascaded genetic and enzymatic reactions, and can mirror real-time biochemical shifts and physiological status within organisms [7]. Metabolomics can directly reflect the results of complex biochemical reaction networks, thereby providing insights into multiple aspects of cell physiology [8]. It is widely applied in fields closely related to human health such as biology, food science, pathology, and toxicology [9]. Beyond caprine and ovine research, metabolomics has been extensively applied to characterize differences in meat quality in cattle, pigs, and chickens, which are core livestock species. For beef cattle, breed-specific lipid and amino acid metabolic fingerprints have been identified to determine marbling tenderness [10]. Indigenous pig lines exhibit distinct metabolic profiles linked to intramuscular fat deposition compared to commercial lean breeds [11]. Poultry metabolomics further reveals breed-driven shifts in flavor precursor accumulation in chicken muscle [12]. In research on sheep, metabolomics provides the conditions for disease diagnosis [13], meat quality analysis [14], performance determination [15], etc. For example, Songjian Yang et al. [16]. found that various metabolites, such as phosphatidic acid (PA), glycerophospholipids, phosphatidylinositol, and phosphatidylcholine, are related to goat meat quality. In addition, Ke Zhang et al. [17] found, based on metabolomics analysis, that breastfeeding and formula feeding caused changes in bile acid and fatty acid metabolism pathways in young goats, further leading to differences in growth rate. Therefore, metabolomic techniques might serve as an effective means of studying differences in meat quality and flavor between local and cultivated goat breeds.

Previous studies have shown that the flavor and quality of chevon are determined by the rich metabolic products in the muscle, such as flavor amino acids, fatty acids, organic acids, peptides, and so on [18]. Similar breed-based metabolic distinctions have been widely reported in pigs, cattle, and sheep: native breeds tend to accumulate metabolites related to stress and disease resistance, while cultivated breeds prioritize rapid-growth metabolic pathways. However, comparable systematic metabolomic comparisons between local and synthetic meat goat lines remain limited. This study took Beichuan White goats and Tianfu goats as the research objects, and untargeted metabolomic techniques were used to analyze the metabolite composition and differential characteristics of the longissimus dorsi muscle (the main edible muscle of goats) between the two breeds. The study hypothesized that distinct breed-specific metabolites in the longissimus dorsi muscle differ significantly between Beichuan White goats and Tianfu goats. These divergent metabolites could regulate chevon quality and flavor formation, and generate correlative metabolic signatures associated with immune regulatory processes. The study aims to characterize the global UHPLC-MS/MS untargeted metabolome of longissimus dorsi muscle from Beichuan White and Tianfu goats, shed light on the metabolic mechanisms underlying their phenotypic divergence, and offer theoretical support for native caprine germplasm conservation, utilization, improvement, and breed-specific feeding regimens.

## 2. Materials and Methods

### 2.1. Ethics Statement

The animal experiments involved in the study were performed in accordance with the method approved by the Ethics Committee of Mianyang Normal University, China (Permit No. SKY101368). All experiments were performed in accordance with relevant guidelines and regulations.

### 2.2. Animals

The study selected 18 1-year-old female goats, with similar body conditions (body weight (Mean ± SD): 30.36 ± 2.13 kg), all of which were healthy and disease-free, and had no-kinship for at least three generations, including 8 Beichuan White goats (BCWLM) and 10 Tianfu goats (TFLM). All goats were raised in the breeding base of Jiyang Livestock Science and Technology Co., Ltd. in Yanting County, Mianyang City, China. The two breeds were raised under identical feeding and management conditions, following the conventional breeding and management program of the breeding base. All goats were provided drinking water ad libitum and fed identical farm-formulated goat total mixed ration (TMR) formulated in accordance with the National Feeding Standard for Meat Goats (NY/T 816-2021). The composition of the basal diet is presented in Table 1. A 30-day pre-experimental feeding period was implemented to eliminate the effects of the pre-experimental environment, followed by a 60-day formal experimental period.

### 2.3. Sample Collection

After the formal feeding period, all goats were fasted for 24 h. The goats were slaughtered with conventional methods. Subsequently, the longissimus dorsi muscle samples were immediately collected and stored in liquid nitrogen in cryovials for subsequent analysis.

### 2.4. Metabolite Extraction and UHPLC-MS/MS Analysis

Metabolism extraction of longissimus dorsi muscle samples was carried out according to the extraction scheme of Fan et al. [19]. Briefly, approximately 50 mg of frozen muscle tissue was weighed and homogenized in pre-cooled methanol–acetonitrile (1:1, *v*/*v*) solvent. Methanol and acetonitrile were purchased from Merck (Darmstadt, Germany). After vortexing, ultrasonic extraction was performed in an ice-water bath using an ultrasonic processor (Scientz, Ningbo, China). The mixture was incubated at −20 °C for protein precipitation and then centrifuged at high speed with an Eppendorf 5810R centrifuge (Eppendorf, Hamburg, Germany). The supernatant was collected, vacuum-dried, and reconstituted with mobile phase solvent prior to LC-MS measurement. LC-MS detection was performed on a Q Exactive mass spectrometer coupled to an UHPLC system (Thermo Fisher Scientific, Waltham, MA, USA). Raw data were processed using Compound Discoverer version 3.3. Meanwhile, equal aliquots from each test sample were pooled to prepare quality control (QC) samples to ensure the reliability of large-scale analysis.

The study used a Vanquish ultra-high-performance liquid chromatography (TUHPLC, Thermo Fisher Scientific, Waltham, MA, USA) system, Chromatographic separation of target metabolites was performed using a Waters ACQUITY UPLC BEH Amide column (Waters Corporation, Milford, MA, USA) (2.1 mm × 50 mm, particle size 1.7 μm) [20]; the mobile phase consisted of phase A (25 mmol/L ammonium acetate + 25 mmol/L ammonia water, pH 9.75) and phase B (acetonitrile). The gradient elution program was set as follows: 0~1 min, 95% B; 1~8 min, 95~65% B; 8~9 min, 65~40% B; 9~10 min, 40% B; 10~11 min, 40~95% B; 11~14 min, 95% B. The column temperature was 30 °C, the flow rate was 0.35 mL/min, the injection volume was 2 μL, and the sample tray temperature was 4 °C. Then, an Orbitrap Exploris 120 mass spectrometer (Thermo Fisher Scientific Co., Ltd., Waltham, MA, USA) was used to collect primary and secondary mass spectrometry data under the control of the control software (Xcalibur, version 4.4, Thermo) (detailed parameters: sheath gas flow rate, 50 Arb; auxiliary gas flow rate, 15 Arb; capillary temperature, 320 °C; full resolution, 60,000; MS/MS resolution, 15,000; collision energy (SNCE), 20/30/40; spray voltage, 3.8 kV (positive) or −3.4 kV (negative)).

### 2.5. Bioinformatics Analysis

The metabolome data were normalized using probabilistic quotient normalization (PQN) before multivariate analysis. Metabolite annotation was performed following the Metabolomics Standards Initiative (MSI) classification criteria. Most metabolites were assigned to MSI level 2 (putatively annotated compounds) based on accurate mass matching and tandem mass spectrum alignment against public databases, with a mass accuracy threshold of 5 ppm. The study used R software (v4.2.3) with the “ropls package” to perform Principal Component Analysis (PCA) and Orthogonal Partial Least Squares Discriminant Analysis (OPLS-DA), followed by a permutation test on the metabolomic data, to identify the overall distribution trends of metabolome and the degree of differences between samples of Beichuan White goats and Tianfu goats. The *t*-test was used to calculate the significance level of metabolites in the longissimus dorsi muscle of the two groups of goats, and the Benjamini–Hochberg false discovery rate (FDR) method was adopted for multiple comparison correction. Metabolites with a VIP > 1, fold change < 0.5 or >2, and adjusted *p* < 0.05 were defined as differentially abundant metabolites [21]. Finally, KEGG functional enrichment analysis of differential metabolites was performed using ‘METABOSIGNAL’ of R (v4.2.3), with the significance threshold set to 0.05.

### 2.6. Correlation Analysis

To determine the relationship between various metabolites, the study utilized R software (v4.2.3) to conduct Pearson correlation analysis on the identified differential metabolites. The correlation analysis results were visualized using R packages “LinkET” and “ggplot2”.

### 2.7. Statistical Analysis

Statistical analysis was conducted using SPSS 27.0 software (IBM, Chicago, IL, USA). Two-sample *t*-tests were applied to compare phenotypic and metabolomic differences between the two goat breeds, with a significance threshold set at *p* < 0.05.

## 3. Results

### 3.1. PCA and OPLS-DA

**Figure 1:**
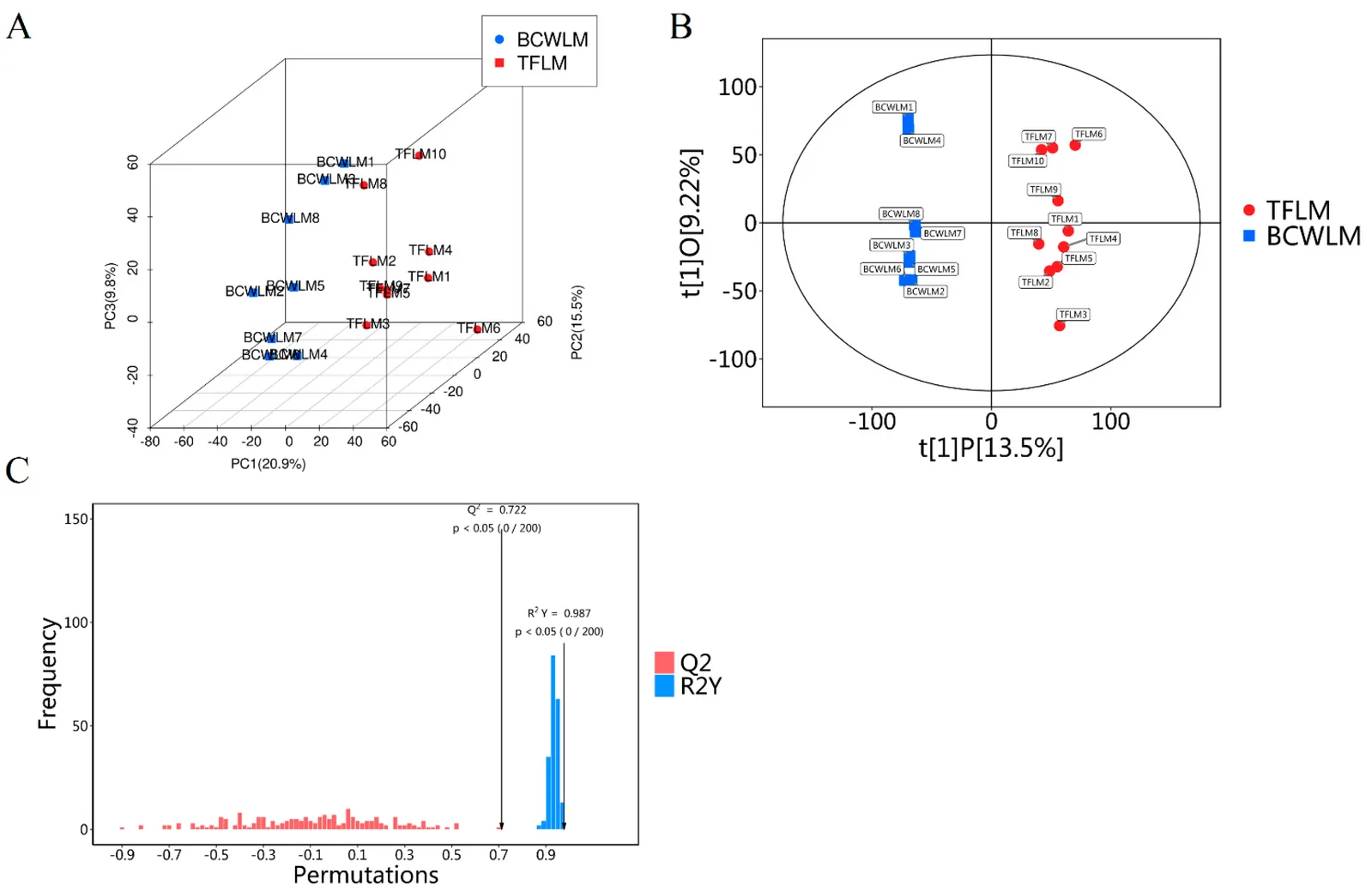
PCA and OPLS-DA of metabolites in longissimus dorsi. (**A**) PCA score graph between BCWLM and TFLM; (**B**) Scatterplot of OPLS-DA model; (**C**) Histogram of permutation test results for the OPLS-DA model.

The PCA results showed that there was a significant separation between the two groups of longissimus dorsi meat samples of Tianfu goats and Beichuan White goats, which reflected divergent metabolite profiles across the breeds. The 3D PCA plot covers three principal components: PC1 explained 20.9% of total variance; PC2 accounted for 15.5%; and PC3 accounted for 9.8% (Figure 1A). Statistical permutation tests validated this inter-group separation. In addition, supervised OPLS-DA was further adopted to quantify breed discrimination and screen key metabolites by VIP values; its model parameters were R2Y = 0.987 and Q2 > 0.5, confirming strong stability and predictive power (Figure 1B,C).

### 3.2. Screening of Differential Metabolites

**Figure 2:**
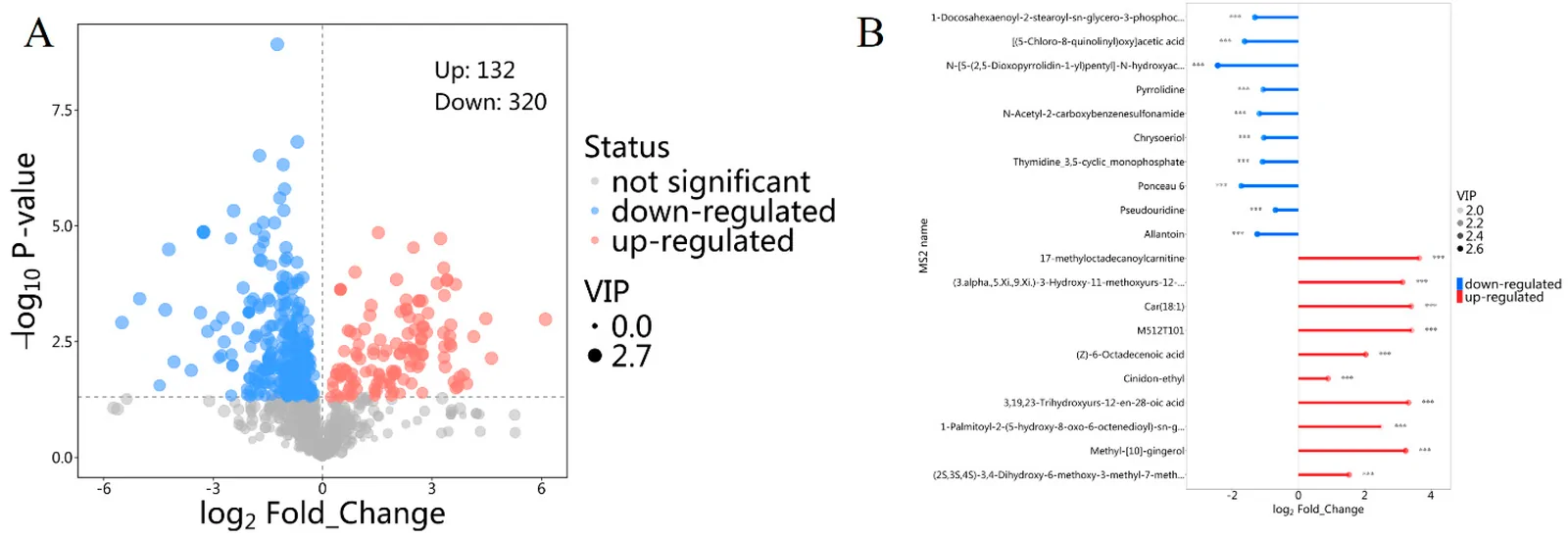
Screening of Differential Metabolites. (**A**) Volcano of metabolites with significant difference; (**B**) A matchstick diagram of difference metabolites.

A total of 452 differential metabolites were screened, including 100 lipids and lipid molecules, 74 organic acids and their derivatives, 12 nucleosides, nucleotides and analogues, 204 organic compounds, and 62 unclassified (Table S1). The volcano plot (Figure 2A) illustrates the global distribution of these metabolites according to log_2_ (fold change) and −log_10_(P), showing that 132 metabolites presented higher abundance in Beichuan White goats (29.20%) and 320 metabolites presented significantly higher abundance in Tianfu goats (70.80%). Figure 2B further presents the top discriminatory metabolites, ranked by variable importance in projection (VIP) values. Nucleosides, nucleotides, and analogues, except N-(2-Furanylmethyl) adenosine, had higher abundance in Beichuan White goats, and 83.78% of organic acids and their derivatives showed higher abundance in Beichuan White goats. No obvious numerical disparity was observed for lipid-derived differential metabolites between breeds, with 52 metabolites of higher abundance in Beichuan White goats and 48 metabolites of higher abundance in Tianfu goats (Figure 2A,B).

### 3.3. Hierarchical Cluster Analysis of Differential Metabolites

**Figure 3:**
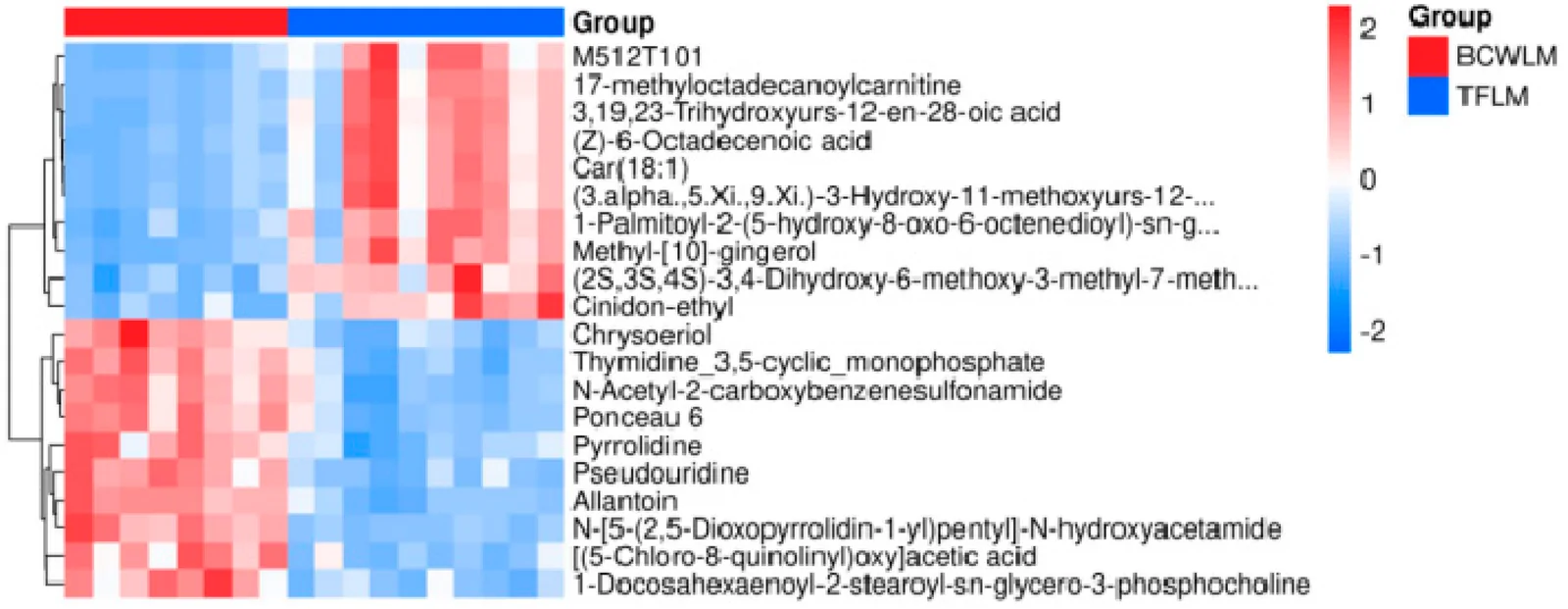
Hierarchical clustering heat map of differential metabolites.

Hierarchical clustering heatmap displays the top 20 most significantly differential metabolites in the TFLM group: M512T101, 17-methyloctadecanoylcarnitine, 3,19,23-Tr-hydroxyurs-12-en-28-oic acid, (Z)-6-Octadecenoic acid, Car (18:1), and other metabolites showed higher abundance, while metabolites such as Chrysoeriol, Thymidine_3, 5-cyclic_monophosphate, N-Acetyl-2-carboxybenzenesulfonamide, Ponceau 6, and Pyrrolidine showed lower abundance (Figure 3).

### 3.4. KEGG Analysis of Differential Metabolites

**Figure 4:**
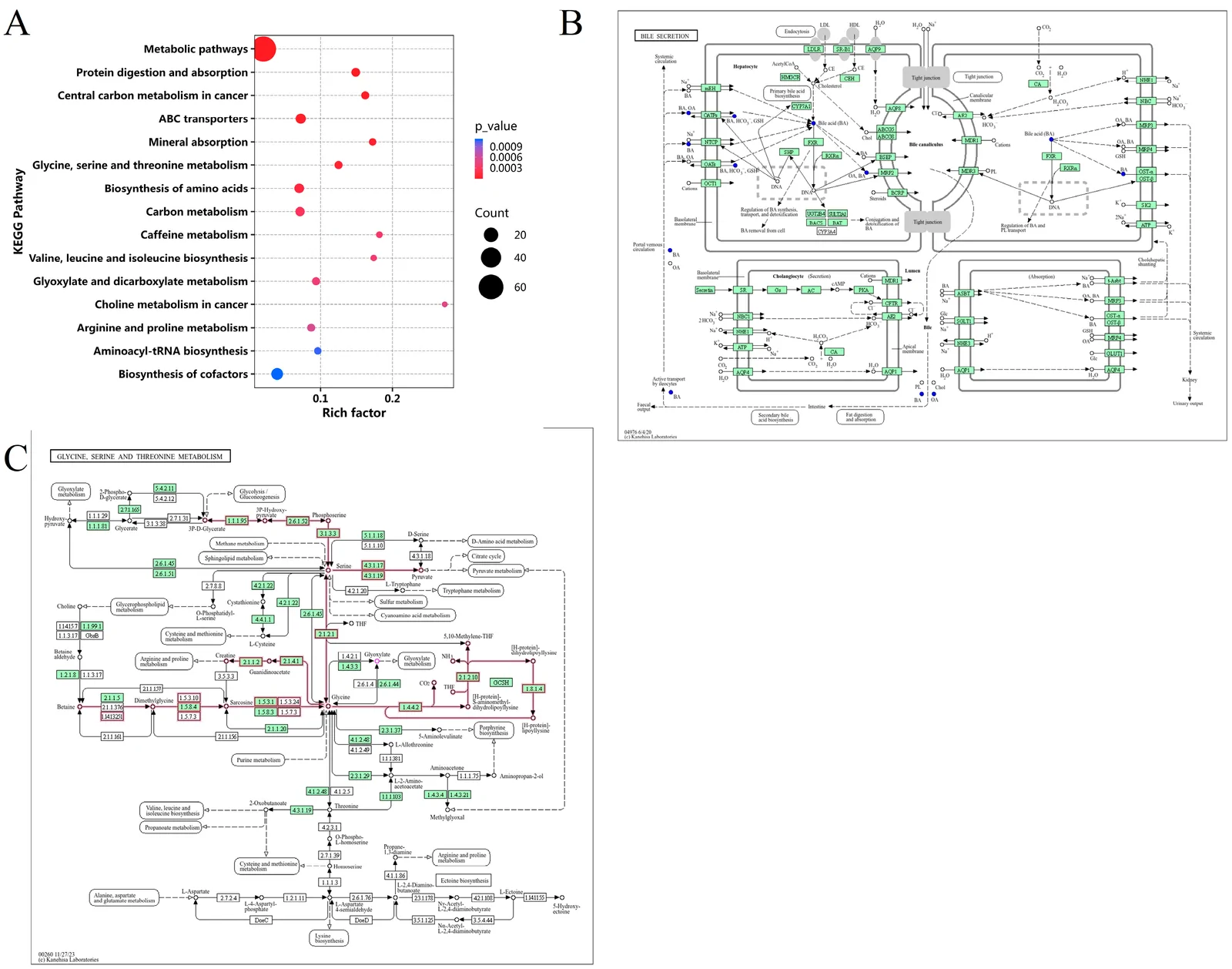
KEGG enrichment analysis of differential metabolites. (**A**) Bubble plot of significant metabolites; (**B**) Bile secretion pathway; (**C**) Glycine, serine, and threonine metabolism.

The results of the KEGG analysis showed that the differential metabolites were significantly enriched in 33 pathways (Table 2, *p* < 0.05). The most significantly enriched pathways were 15 pathways including metabolic pathways, protein digestion and absorption, and central carbon metabolism in cancer (Figure 4A). Notably, in the bile secretion, Pregnanediol 3-O-glucuronide and chenodeoxycholic acid showed lower abundance in Tianfu goats compared with BCWG (Figure 4B). In the glycine, serine, and threonine metabolism, betaine, glycine, sarcosine, and glyoxylic acid all presented lower abundance in TFLM (Figure 4C).

### 3.5. Correlation Among Differentially Abundant Metabolites

**Figure 5:**
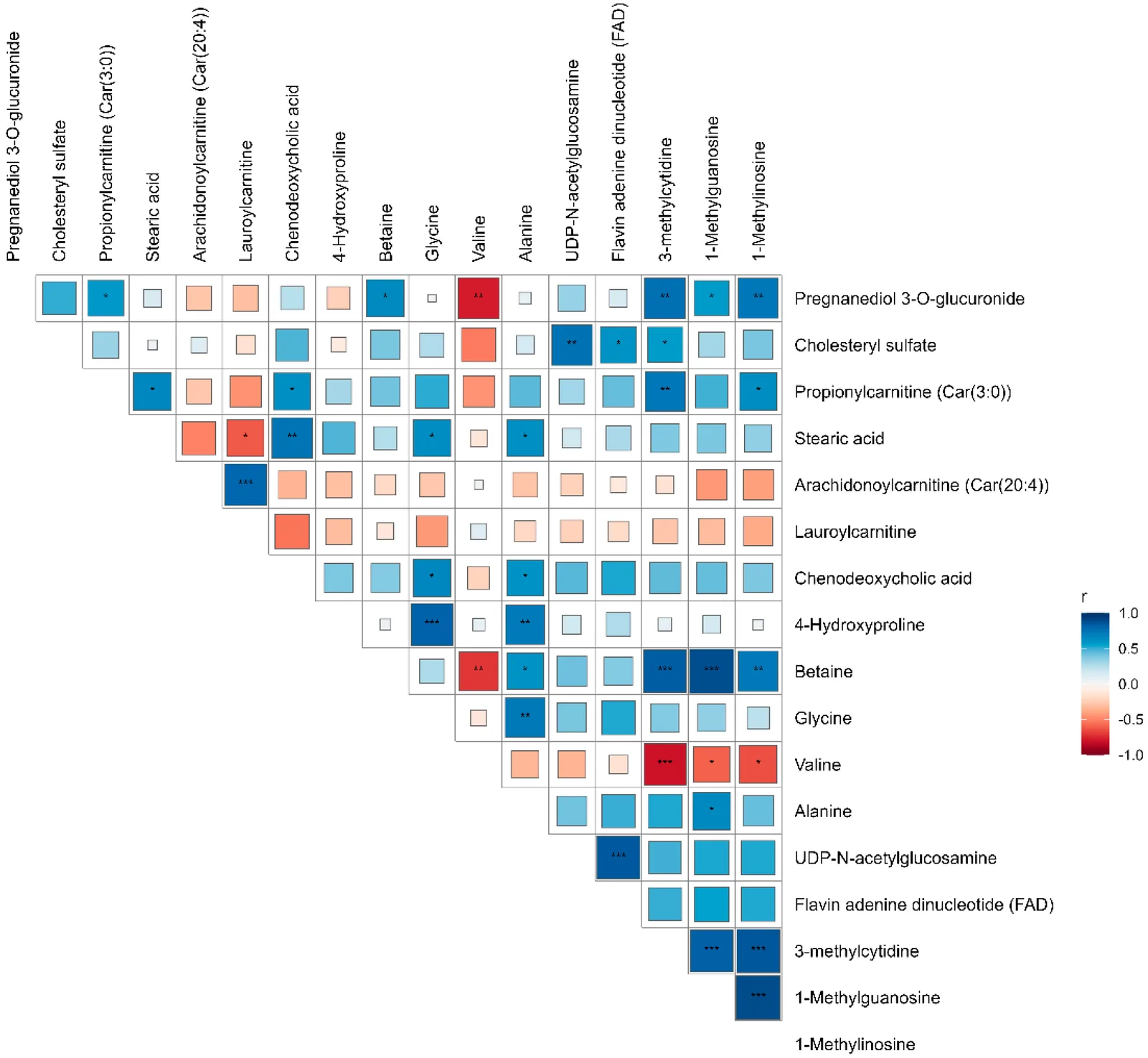
Correlation analysis of differential metabolites. Asterisks indicate significance levels: * *p* < 0.05, ** *p* < 0.01, *** *p* < 0.001.

To further elucidate the synergy of changes between metabolites, Pearson correlation coefficient analysis was performed. The results showed that there was a significant positive correlation between 1-Methylguanosine and 1-Methylinosine, 3-methylcytidine, Betaine, Flavin adenine dinucleotide (FAD), Pregnanediol 3-O-glucuronide, Propionylcarnitine (Car (3:0)), UDP-N-acetylglucosamine, and a significant negative correlation between 1-Methylguanosine, 1-Methylinosine, 3-Methylcytidine and Valine, 4-Hydroxyproline, Alanine, and N-(2-Furanylmethyl) adenosine (Figure 5).

## 4. Discussion

The study was based on untargeted metabolomics analysis of significant differences in metabolite composition between the longissimus dorsi of Beichuan White goats and Tianfu goats, further elucidating the potential impact of these differences on their meat quality and physiology. With the increasing consumer demand for high-quality, healthy lamb meat, the research not only has theoretical value but also provides a scientific basis for the utilization of local goat breeds and the improvement of goat breeds.

PCA and OPLS-DA both showed significant metabolic differences between the two groups of goats, indicating that the metabolic activity in the animal’s body is influenced by its breed type and feeding environment [22]. The Beichuan White goat is famous for its high-quality meat and unique flavor, while Tianfu goats have the advantages of a tall body and fast growth and development. The metabolic basis behind these phenotypic characteristics was discussed in this study.

Lipids act as primary flavor precursors for cooked chevon, as their thermal oxidation during processing generates abundant volatile aromatic substances including aldehydes, ketones, and furans, which dominate the lamb’s unique sensory profile [23]. It is well known that lipids are important flavor precursors in chevon. In this study, a total of 100 lipid and lipid molecule differential metabolites were identified between the two goat breeds, of which 52 showed higher abundance in Beichuan White goats (Figure 2), including Propionylcarnitine, stearic acid, and cholesteryl sulfate and other key lipid metabolites. Propionylcarnitine is an acylcarnitine metabolite, which is related to fatty acid oxidation and energy metabolism [24,25,26]. The higher abundance of Propionylcarnitine in Beichuan White goats indicated that the fatty acid oxidation metabolism of Beichuan White goat muscle tissue was more active, which could provide sufficient energy for muscle cell metabolism and affect the deposition of intramuscular fat. Stearic acid, as a precursor of certain essential flavor compounds in lamb, is closely related to the aroma intensity of lamb [27]. Notably, the content of stearic acid in lamb could affect the melting point of intermuscular fat and the juiciness of meat [28]. Its higher abundance in Beichuan White goats serves as an important metabolic basis for superior meat flavor and juiciness compared with Tianfu goats. Cholesteryl Sulfate is related to the biosynthesis of cholesterol [29], and its higher abundance in Beichuan White goats is conducive to the synthesis of cholesterol in meat tissue, which further affects the metabolism of fatty acids and the formation of meat flavor. In contrast, Tianfu goats showed a higher abundance of Arachidonoylcarnitine and Lauroylcarnitine (Figure 3), which are lipid metabolites linking lipid turnover to inflammatory immune responses. Lauroylcarnitine drives T-cell secretion of proinflammatory cytokines, while arachidonoylcarnitine modulates metabolic homeostasis that shapes immune status, as supported by cited references. Such breed-specific shifts in lipid metabolism constitute a key driver of divergent meat flavor between Beichuan White goats and Tianfu goats. Additionally, Chenodeoxycholic acid has many functions, such as anti-oxidation, regulation of apoptosis and glucose and lipid metabolism, and enhancement of immunity [30,31,32]. Arachidonoylcarnitine plays a role in glucose metabolism and cardiovascular function [33], and Lauroylcarnitine might be a molecular marker of inflammation, which could promote the secretion of proinflammatory cytokines by T cells [34,35]. The levels of Arachidonoylcarnitine and Lauroylcarnitine in the longissimus dorsi meat of Tianfu goats were higher, which suggests a potential relatively weaker immune trait in Tianfu goats compared with Beichuan White goats. This was also related to its breed characteristics of fast growth and high lean meat rate.

The content and composition of amino acids are important indicators for evaluating the flavor of mutton [36]. This study found that 4-hydroxyproline, betaine, alanine, and glycine all showed higher abundances in the longissimus dorsi muscle of Beichuan White goat. 4-Hydroxyproline is a special amino acid used to measure collagen content, whose content and properties can greatly affect the sensory and edible quality of meat products [37]. Betaine could not only prevent lipid peroxidation and improve growth performance and meat quality [38,39], but also could be used as a feed additive to selectively increase the content of intramuscular fat in pigs [40]. Alanine (Ala) and glycine (Gly) are the major contributors for taste [41].

KEGG enrichment analysis showed that 4-hydroxyproline, glycine, valine, alanine, and keucine were significantly enriched in the ABC transporters pathway, and glycine was also significantly enriched in the glycine, serine, and threonine metabolism. The ABC transporters use ATP binding and hydrolysis to drive the transmembrane transport of nutrients such as lipids, organic acids, and their derivatives [42,43]. The glycine, serine, and threonine metabolism pathway has been proved to be the key metabolic pathway for the formation of chicken flavor [44]. This study speculated that amino acids and their derivatives might regulate the flavor changes of longissimus dorsi muscle by participating in ABC transporters and the glycine, serine, and threonine metabolism. In addition, amino acids are essential substances for the survival of the body; their main functions include physiological regulation, enhancing immunity, maintaining nutritional homeostasis, and providing energy. In recent years, the use of low-cost crystalline amino acids or animal protein feed substitutes to develop low-protein diets and achieve precise nutrition has attracted considerable attention. This requires a better understanding of the nutritional requirements and metabolic patterns of amino acids in livestock and poultry [45].

A total of 13 differential metabolites of nucleosides and nucleotides were screened in this study. UDP-N-acetylglucosamine is a very important member of glyconucleotides, which could participate in the synthesis of human milk oligosaccharides (HMOs), N-glycans, O-glycans, and proteoglycans in glycoproteins, and is closely related to the immune regulation and neural development of the body [46,47,48]. FAD provides a large amount of ATP for the cellular immune response process, as an important cofactor of energy metabolism [49,50]. 1-Methylinosine, 1-Methylguanosine, and 3-methylcytidine are a type of RNA methylation [51], which plays a vital role in innate and adaptive immune responses, affecting macrophage polarization, B-cell proliferation, T-cell infiltration and activation, and other biological processes [52,53]. Only N-(2-Furanylmethyl) adenosine showed a higher abundance in the longissimus dorsi meat of Tianfu goats. As a potent immunomodulatory molecule, adenosine exerts immunosuppressive effects and inhibits the activation of immune cells, thereby playing a vital role in innate and adaptive immune responses, affecting macrophage polarization, B-cell proliferation, T-cell infiltration and activation, and other biological processes [54].

Additionally, most of the above metabolites were enriched in the metabolic pathway. UDP-N-acetylglucosamine and Uridine 5′-diphosphoglucuronic acid (UDP-glucuronate) were enriched in the biosynthesis of nucleotide sugars, and pseudouridine was enriched in the pyrimidine metabolism. Previous research has shown that nucleoside sugar is the cornerstone of carbohydrate and its complex biosynthesis [55], which plays an important role in signal transduction through post-translational modifications of proteins [56]. Flavin adenine dinucleotide (FAD) was significantly enriched in the biosynthesis of cofactors, riboflavin metabolism, and vitamin digestion and absorption pathways. Riboflavin and its derivative FAD play a crucial role in important cellular processes such as mitochondrial energy metabolism, stress responses, and the regulation of vitamins and cofactor biological genes [50,57]. Vitamins were essential nutrients for the growth and development of the body and the realization of immune function [58,59], which is one of the important reasons for the relatively weak immune capacity of Tianfu goats compared with Beichuan White goats. It is worth noting that the bile secretion pathway was also significantly enriched in the differential metabolites (Table 2, Figure 4B). Pregnanediol 3-O-glucuronide and chenodeoxycholic acid showed higher abundances in the Beichuan White goat, and were significantly enriched in bile secretion. Bile secretion mainly produces bile acid, which is not only an important factor in the regulation of glucose, fat, and energy metabolism [60], but could also promote the expression of steroidogenesis-related genes in adrenal cortical cells by increasing the phosphorylation of extracellular signal-regulated kinase (ERK), activating S1PR2 and steroidogenic factor SF-1, significantly increasing the secretion level of cortisol, and improving the anti-inflammatory ability of the body [61,62,63].

This study detected an extensive metabolomic difference between Beichuan White and Tianfu goats, which is attributed to breed-specific physiological traits. Multiple key limitations exist, however. First, we lacked SNP, InDel, or whole-genome sequencing evidence to confirm inherent physiological differences between the two Sichuan goat breeds, and no relevant published data could link differential metabolites to causal molecular loci. Second, all flavor and juiciness deductions relied purely on untargeted metabolomics without supporting meat phenotype measurements (pH, shear force, IMF, sensory evaluation, volatile compounds, etc.). Third, only female goats were sampled with unbalanced group sizes (8 vs. 10), and no pre-experiment power analysis was conducted, restricting result generalizability. Additionally, bile acid disparities only tentatively imply stronger disease resistance in Beichuan White goats, requiring further immunological verification. Future multiomic studies combining whole-genome sequencing and metabolomics are needed to unravel the underlying basis of breed-specific meat quality and immune metabolic phenotypes.

Overall, differential metabolites such as lipids, amino acids, organic acids and their derivatives, and nucleotides might correlate with interbreed divergence in chevon flavor and meat quality; meanwhile, the observed nucleotide disparities merely indicate a possible association with distinct disease-resistance traits, without supporting immune-related experimental data. It is acknowledged that relying solely on metabolomics is insufficient to comprehensively elucidate the breed-specific mechanisms in meat goats. In subsequent research, we plan to combine transcriptomics, proteomics, and other omics techniques with goat phenotypic data to further elucidate the differences between local goat breeds and cultivars in a more in-depth and comprehensive manner.

## 5. Conclusions

The study characterized the longissimus dorsi muscle metabolome of Beichuan White goats and Tianfu goats via untargeted metabolomics. In total, 452 differential metabolites were identified: 132 metabolites showed higher abundance in Beichuan White goats and 320 metabolites presented higher abundance in Tianfu goats. Differential lipids and amino acids (propionylcarnitine, stearic acid, cholesteryl sulfate, carnitine, valine, etc.) may act as key metabolic candidates underlying interbreed differences in muscle characteristics and chevon flavor. The higher abundance of nucleoside and nucleotide metabolites in Beichuan White goats potentially enhances disease resistance, and these metabolites may modulate meat quality via ABC transporters as well as the glycine, serine, and threonine metabolism. UDP-N-acetylglucosamine, 1-methylinosine, 1-methylguanosine, 3-methylcytidine, and N-(2-furanylmethyl) adenosine are potential metabolic correlates that might be linked to probable differences in disease resistance between breeds, yet definitive immune verification is still required in follow-up research.

## Figures and Tables

**Table 1:** Basic diet composition and nutrition level of goats.

Composition of Feed Raw Materials	Composition (%)	Nutrition Level	Composition
Maize	39.00	Metabolizable Energy ^2^ (MJ/kg)	9.30
Alfalfa	6.00	Crude Protein (CP, %)	13.52
Mill offals	12.00	Neutral Detergent Fiber (NDF, %)	45.73
Corn for silage	18.00	Acid Detergent Fiber (ADF, %)	32.39
Corn stalk	22.00	Calcium (Ca, %)	0.56
Premix ^1^	3.00	Phosphorus (P, total, %)	0.38
Total	100.00		

Note: ^1^ Each kilogram of premix contains vitamin A 150,000 IU/kg, vitamin D 380,000 IU/kg, vitamin E 200 IU/kg, nicotinamide 334 mg/kg, copper 160 mg/kg, cobalt 25 mg/kg, manganese 500 mg/kg, iron 600 mg/kg, iodine 6 mg/kg, zinc 800 mg/kg, and selenium 2 mg/kg. ^2^ Metabolic Energy in the nutritional level is the calculated value, and the rest is the measured value.

**Table 2:** The results of KEGG enrichment analysis of differential metabolites (*p* < 0.05).

Pathway	Counts	Rich_Factor	*p*_Value
Metabolic pathways	63	0.02049447	2.03 × 10^−6^
Protein digestion and absorption	7	0.14893617	5.41 × 10^−6^
Central carbon metabolism in cancer	6	0.162162162	1.61 × 10^−5^
ABC transporters	10	0.072463768	3.63 × 10^−5^
Mineral absorption	5	0.172413793	6.34 × 10^−5^
Glycine, serine and threonine metabolism	6	0.125	7.43 × 10^−5^
Biosynthesis of amino acids	9	0.0703125	0.000116896
Carbon metabolism	8	0.071428571	0.000258698
Caffeine metabolism	4	0.181818182	0.000293533
Valine, leucine and isoleucine biosynthesis	4	0.173913043	0.000351271
Glyoxylate and dicarboxylate metabolism	6	0.09375	0.000375103
Choline metabolism in cancer	3	0.272727273	0.000511913
Arginine and proline metabolism	6	0.086956522	0.000564998
Aminoacyl-tRNA biosynthesis	5	0.096153846	0.001057814
Biosynthesis of cofactors	13	0.039634146	0.001115612
Insulin resistance	3	0.157894737	0.002754828
Valine, leucine and isoleucine degradation	4	0.095238095	0.003572262
2-Oxocarboxylic acid metabolism	7	0.048611111	0.00588734
Amino sugar and nucleotide sugar metabolism	6	0.050847458	0.008667991
Glycerophospholipid metabolism	4	0.071428571	0.009995892
Pantothenate and CoA biosynthesis	3	0.1	0.010242978
Biosynthesis of nucleotide sugars	8	0.04	0.010358876
Sulfur relay system	2	0.181818182	0.011518652
Pentose and glucuronate interconversions	4	0.06779661	0.011972021
beta-Alanine metabolism	3	0.09375	0.012245857
Sphingolipid metabolism	3	0.085714286	0.015644104
Pyrimidine metabolism	4	0.0625	0.015793499
Pentose phosphate pathway	3	0.081081081	0.018176055
Glycerolipid metabolism	3	0.078947368	0.01952264
D-Amino acid metabolism	4	0.057971014	0.020308371
Diabetic cardiomyopathy	3	0.076923077	0.020923151
Sphingolipid signaling pathway	2	0.133333333	0.021144818
Lipoic acid metabolism	3	0.06181818	0.028736005

## Data Availability

All the figures and tables used to support the results of this study are included.

## Supplementary Materials

The following supporting information can be downloaded at: https://www.mdpi.com/article/10.3390/metabo16080537/s1; Table S1: Differential metabolites in longissimus dorsi meat of Beichuan White goat and Tianfu goat.
